# Slip and Magnetic Attraction Effects in a Microrobot with Magnetic-Wheels and Skid-Steering

**DOI:** 10.3390/mi10060379

**Published:** 2019-06-06

**Authors:** Munehisa Takeda, Isao Shimoyama

**Affiliations:** 1 MEMS System Development Center, Micromachine Center, Tokyo 101-0026, Japan; 2 Toyama Prefectural University, Imizu 939-0398, Japan; i-shimoyama@pu-toyama.ac.jp or isao@leopard.t.u-tokyo.ac.jp; 3 Graduate School of Information Science and Technology, The University of Tokyo, Tokyo 113-8656, Japan

**Keywords:** microrobot, magnetic wheel, slip, magnetic attraction, turning characteristics, skid-steered

## Abstract

This study investigated slip and magnetic attraction effects in a skid-steered magnetic-wheeled microrobot. The dynamics of the microrobot were derived by considering the slip and magnetic attraction of the wheels. In addition, the slip characteristics of the magnetic wheels were measured using an evaluation apparatus built for this purpose. A simulation program for driving performance was developed as well. Simulations indicated that the turning characteristics of the skid-steered wheeled microrobot degrade because the gripping force decreases due to the decrease in weight with decreasing size. However, the turning characteristics of a skid-steered microrobot can be improved with the magnetic attraction of magnetic wheels. A 5 mm × 9 mm × 6.5 mm skid-steered microrobot with four magnetic wheels was fabricated, and the measured performance was consistent with the simulation results. The differences in driving performance were clarified between a microrobot with column-type magnetic wheels and one with barrel-type magnetic wheels, as well as between forward and backward motion.

## 1. Introduction

Skid-steered wheeled locomotion is suitable for a microrobot because it has a simple structure and can achieve high speeds. However, few microrobots have adopted the method because slippage makes it difficult to ensure sufficient gripping force and control the interaction between the wheels and the ground. We used magnetic wheels and investigated the slip and magnetic attraction effect between the magnetic wheels and ground to achieve a skid-steered wheeled microrobot.

In the macro world, wheeled locomotion modes, such as the automobile and railway, have become mainstream because wheeled transport is the most efficient method, and it enables high-speed movement. However, for micro-world locomotion, various methods in addition to wheels are possible, such as inchworm methods [1], vibration methods [2,3], magnetic attraction methods [4,5,6], and legged methods [7]. This variety is possible because surface forces, such as friction forces, vibration and magnetic attraction, which are not used in the macro world, can be efficiently exploited in the micro-world because of the lighter weight of the mechanisms. However, the wheeled method remains the most promising because it has a simple structure and can achieve high speeds. Therefore, various wheeled microrobots have been developed. Dario et al. [8] developed a microrobot with wobble motors. Caprari et al. [9] developed a microrobot called Alice with dimensions of 21 mm × 21 mm × 18 mm as a research and development platform. Kim et al. [10] developed a millirobot (mROBerTO) for swarm studies.

The driving performance of a wheeled microrobot degrades because of the reduced gripping force and slip between the wheels and ground resulting from the decrease in weight as size decreases. Therefore, Jin et al. [11] and Tang et al. [12] developed omni-directional microrobots with magnetic wheels. We also developed a microrobot with magnetic wheels [13]. Use of magnet attraction is important to make up for a loss of power due to the small volume of the microrobot.

Moreover, design of an uncomplicated structure is also important for a microrobot. For a wheel-driving mechanism, there are four feasible methods: a skid-steered method, a steering method, a caster method, and a method with four independently-driven wheels. The skid-steered method, which has no complicated steering or caster mechanism and fewer motors, is the simplest and most robust structure. Therefore, it is the most suitable method for a microrobot because of its simple mechanism. The skid-steered method is also frequently used for macro mobile robots [14,15]. However, in the skid-steered method, turning is achieved by skidding by driving the left and right wheels at differing speeds. Therefore, it is important to have enough gripping force between the wheels and ground and to understand the slip characteristics of the wheels.

Studies have investigated the driving performance of large mobile robots, including the slip of the tires [16,17,18,19,20]. However, few studies have focused on the driving performance of microrobots by studying the slip and gripping force between the wheels and ground. In order to realize the skid-steered type microrobot, it is important to understand the slip characteristics of the wheels and the ground and to clarify the effect of the magnet attraction. If a skid-steered type microrobot can be realized, it will be possible to apply them to inspection, cleaning, transportation, security, and environmental fields in narrow places, such as inside pipes and various equipment.

This study reports the dynamics of a skid-steered wheeled microrobot, including the slip and magnetic attraction of magnetic wheels, the results of measurements of the slip characteristics of the magnetic wheels, and an analysis of the turning characteristics of a microrobot with magnetic wheels. Additionally, we report the outline of a prototype skid-steered magnetic-wheeled microrobot of dimensions 5 mm × 9 mm × 6.5 mm and the experimental results of its driving performance.

## 2. Dynamics of a Skid-Steered Wheeled Microrobot

### 2.1. Kinematic Theory of a Skid-Steered Wheeled Microrobot

**Figure 1:**
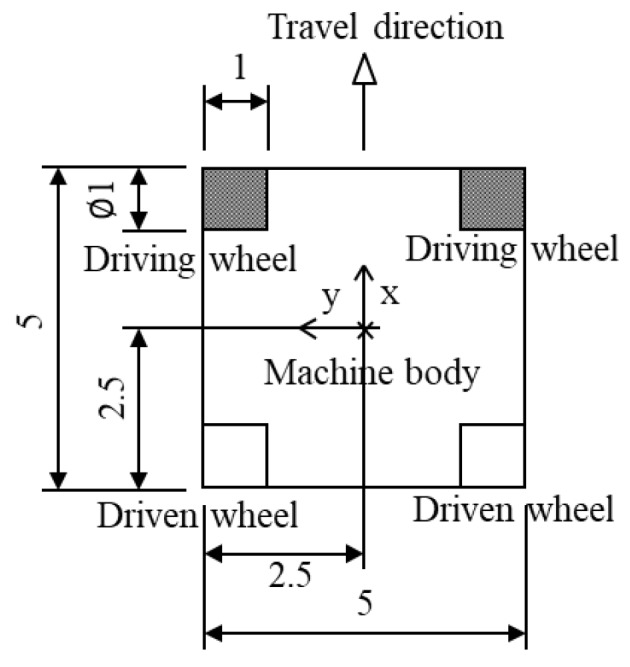
A four-wheeled model. Four wheels are located at the corners of the machine body. Front gray wheels show driving wheels and rear white wheels show driven wheels. The machine is assumed to be a cube of 5 mm on a side, and the wheel is a cylinder of 1 mm in diameter and 1 mm in length.

We derived the dynamics of a skid-steered wheeled microrobot from the kinematic theory of automobiles and manipulators. A skid-steered wheeled microrobot is composed of a body and four wheels, as shown in Figure 1.

The skid-steered method causes the microrobot to turn using a speed difference between the left and right driving wheels (front wheels), which are fixed and cannot turn sideways; the method is used for steering in the horizontal plane.

The kinematic theory of such a microrobot is identical to that of an automobile: the motion equations are derived in a coordinate system fixed on the moving body, and the two-dimensional movements from friction between the wheels and ground are obtained. However, the kinematic theory of a skid-steered wheeled microrobot is different from that of automobiles in terms of the required consideration of slip. Slip occurs laterally when the microrobot turns. Therefore, the movement of the microrobot does not obey the so-called "Ackerman geometry" framework, which is the base of the kinematic theory of automobiles and determines the turning radius by the speed difference of the wheels and their geometric relationship.

A skid-steered wheeled microrobot follows the same kinematic theory as manipulators in the formulation of a multi-link, in which a rigid link connects to a wheel with a joint. However, the kinematic theory of the microrobot differs from that of manipulators because there are multiple ends for the base and no fixed point for a standard coordinate system.

Developing the motion equations of a skid-steered wheeled microrobot based on the above kinematic theory, we derived the following equation from the balance equations of all external forces that act on the base:

(1) $$m_{{\bar{D}}_{3}}{\ddot{p}}_{3}+{\dot{\omega }}_{3}\underset{j\in D_{3}}{\sum }m_{j}p_{j3}+\omega_{3}\times \left(\omega_{3}\times \underset{j\in D_{3}}{\sum }m_{j}p_{j3}\right)-{\bar{f}}_{{\bar{D}}_{3}}=0$$

where

$m_{j}$: mass of link *j*, $m_{{\bar{D}}_{3}}$: all mass of body and wheels,
$p_{j}$: position of link *j*,
$\omega_{j}$: angular velocity of link *j*,
${\bar{f}}_{{\bar{D}}_{3}}$: sum of the forces acting on the wheels from the ground.

In addition, the following equation is derived from the balance equations of all moments that act on the base.

(2) $$\begin{array}{l} \underset{j\in {\bar{D}}_{3}}{\sum }\left(I_{j}{\dot{\omega }}_{j}+\omega_{j}\times I_{j}\omega_{j}\right)+\underset{j\in C_{3}}{\sum }m_{j}p_{j3}\times {\ddot{p}}_{3}+\underset{j\in C_{3}}{\sum }m_{j}p_{j3}\times \left({\dot{\omega }}_{3}\times p_{i3}\right)\\ \;\;\;\;\;\;+\underset{j\in C_{3}}{\sum }m_{j}p_{j3}\times \left\{\omega_{3}\times \left(\omega_{3}\times p_{i3}\right)\right\}-\underset{j\in C_{3}}{\sum }p_{j3}\times {\bar{f}}_{j}-\underset{j\in C_{3}}{\sum }{\bar{n}}_{j}=\;0 \end{array}$$

where

$I_{j}$: inertial moment of link *j*,
${\bar{n}}_{j}$: sum of moments acting on the wheels from the ground.

### 2.2. Friction Force between the Wheel and Ground

#### 2.2.1. Wheel Coordinate System

**Figure 2:**
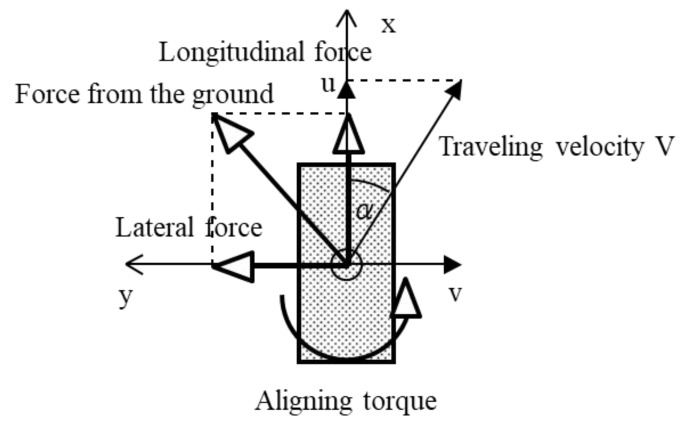
Forces to act on a wheel. The “longitudinal force”, “lateral force”, and “aligning torque” indicate the friction acting on the x- and y-axes and the friction moment around the z-axis, respectively.

The wheel coordinate system is defined as the x-axis in the wheel longitudinal direction, as shown in Figure 2. Figure 2 also shows the “longitudinal force”, “lateral force”, and “aligning torque”, which indicate the friction acting on the x- and y-axes and the friction moment around the z-axis, respectively.

The slip ratio ($s_{wi}$) and slip angle ($\alpha_{wi}$) of wheel wi are defined as follows:

(3) $$s_{wi}=\frac{u_{wi}-{\bar{r}}_{wi}\cdot {\dot{\emptyset }}_{wi}}{u_{wi}}\;\alpha_{wi}=\tan^{-1}\frac{v_{wi}}{u_{wi}}$$

where

${\bar{r}}_{wi}$: radius of wheel *i*,
$u_{wi}$: x-axis directional speed of wheel *i*,
${\dot{\emptyset }}_{wi}$, rotating speed of wheel *i*,
$v_{wi}$: y-axis directional speed of wheel *i*.

#### 2.2.2. Model of Tire Slip Characteristics

We first describe a model of tire slip characteristics for which the relationship between the slip and friction force is clear. A model is based on the Magic Formula [21] developed by the group of Professor Pacejka of Delft University in The Netherlands. The Magic Formula is commonly used as a tire slip model because the numerical formula is relatively simple and fitting it to experimental data is also simple. The total amount of slip (*σ*) is derived from the slip ratio (*s*) and slip angle (*α*) in Equation (4).

(4) σ=sin2α+(KxKy·s·cosα)2

where

*K_x_*: driving stiffness,
*K_y_*: cornering stiffness.

The coefficient of friction μ will decrease based on σ according to:

(5) $$\mu =\mu_{0}\left(1-K_{\mu }\cdot \sigma \right)$$

where

*μ*_0_: maximum coefficient of static friction,
*K_u_*: decrease in the coefficient when the friction changes from static friction to dynamic friction (= 0.2).

The characteristic function of a tire is defined as follows:

(6) $$f\left(\sigma ,f_{z}\right)=\mu \cdot f_{z}\cdot \sin\left(a\cdot \tan^{-1}\left(b\cdot \sigma \right)\right)$$

where

a: constant to determine the form of the function (= 1.2), $b=\frac{K_{y}}{\mu_{0}\cdot f_{z}}$

Finally, the longitudinal force (*f_x_*) and lateral force (*f_y_*) are expressed in the following equations as functions of *s* and *α* of Equation (3), and z-axis force (*f_z_*):

(7) $${\bar{f}}_{x}\left(s,a,f_{z}\right)=-\;\frac{K_{x}}{K_{y}}\cdot \frac{s\cdot |\cos\alpha |}{\sigma }\cdot \;f\left(\sigma ,f_{z}\right)\;{\bar{f}}_{y}\left(s,a,f_{z}\right)=-\;\frac{\sin\alpha }{\sigma }\cdot \;f\left(\sigma ,f_{z}\right)$$

**Figure 3:**
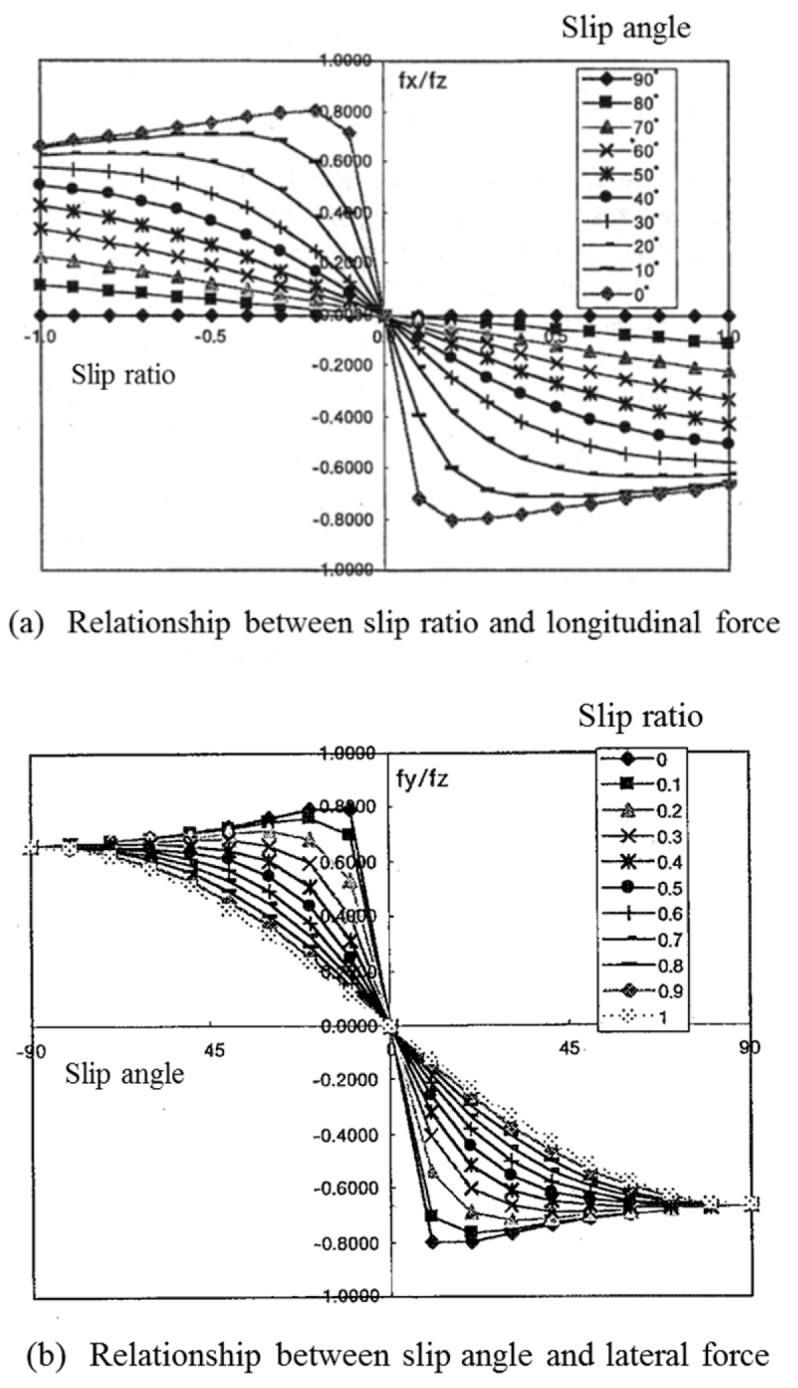
Slip characteristics of tire. (**a**) Relationship between slip ratio and longitudinal force where slip angle is changed from 0 degree to 90 degrees. (**b**) Relationship between slip ratio and lateral force where slip ratio is changed from 0 to 1.

We assume that the weight of a microrobot is equally distributed on the four wheels, the force in the z direction on a wheel is *f_z_* and that the driving stiffness *K_x_* and cornering stiffness *K_y_* are related by Equation (8). Then the slip characteristics of the tire, as shown in Figure 3a,b, are provided as functions of the friction force and slip of Equation (7).

*K_x_* = *K_y_* = 10 × *f_z_.* (8)

Figure 3a,b show the slip characteristics of the longitudinal and lateral directions, respectively. They are experimental identification models. Depending on the nonlinearity characteristics of the tire rubber, the longitudinal force decreases in slip angle 0 degree of Figure 3a when the slip ratio is larger than 0.2, and the lateral force decreases in slip ratio 0 of Figure 3b when the slip angle is larger than 10 degrees.

## 3. Measurement of the Slip Characteristics of a Magnetic Wheel

### 3.1. Apparatus for Evaluation of the Characteristics of a Magnetic Wheel

**Figure 4:**
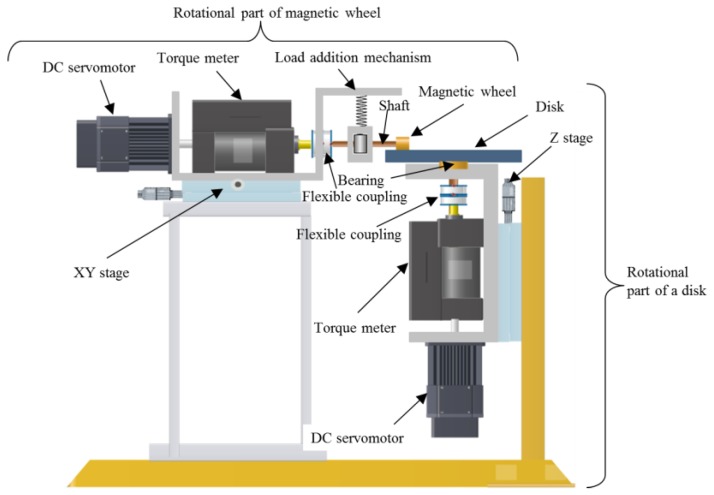
Characteristics evaluation apparatus for magnetic wheel. The apparatus consisted of a rotational part of a magnetic wheel and a rotational part of a disk.

It is difficult to measure the slip characteristics of 1 mm diameter magnetic wheels that are installed in an actual microrobot because of the limited range of measurement of a torque meter. An apparatus was developed for evaluation of slip characteristics of magnetic wheels of 8 mm diameter. Torque meters with the smallest commercially available measurement range were installed in the apparatus. The apparatus is shown in Figure 4 and consisted of a rotational part of a magnetic wheel and a rotational part of a disk.

On the tip of the rotational part of the magnetic wheel, a magnetic wheel with a diameter of 8 mm was connected to a torque meter through a shaft and flexible coupling. A DC servomotor was connected to the torque meter. Therefore, the magnetic wheel could be rotated with any rotational frequency. Furthermore, a load-addition mechanism was attached to the upper part of the shaft. The load-addition mechanism and flexible coupling absorbed the eccentricity of the magnetic wheel. The magnetic wheel was fixed to the shaft by a screw and could be changed. In addition, the rotational part of the magnetic wheel was attached to an XY stage that could fix the wheel at any position in the horizontal plane.

**Figure 5:**
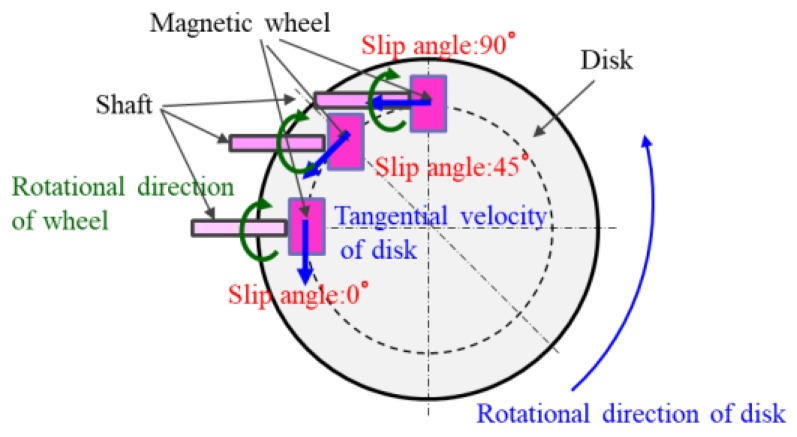
Arrangement of a magnetic wheel on a disk. When the slip angle was 0, the magnet wheel was installed so that the rotation direction of the magnet wheel and the tangential direction of the disk were in the same direction. When the slip angle was 90 degrees, the magnet wheel was installed so that the rotation direction of the magnet wheel and the tangential direction of the disk became 90 degrees.

For the rotational part of the disk, a disk was connected to a torque meter through a bearing and coupling. Similar to the rotational part of the magnetic wheel, the disk can be rotated with any frequency by a DC servomotor. In addition, the rotational part of the disk was attached to the Z stage and could vertically adjust the bottom of a magnetic wheel to a position on the disk’s top surface. The longitudinal force *f_x_* and lateral force *f_y_* of a magnetic wheel at an arbitrary slip ratio and slip angle can be calculated by the following Equation (9). The torque values of *Tw* and *TD* in Equation (9) were measured by the torque meters of the rotational part of the magnetic wheel and the rotational part of the disk, respectively, while controlling the rotational frequency of the magnetic wheel and disk, and by fixing a magnetic wheel to the predetermined position on the disk shown in Figure 5.

*Tw* = *r*·*f_x_* *TD* = *R*(*f_x_*cos*α* − *f_y_*sin*α*) *f_x_* = *Tw*/*r* *f_y_* = cosec*α*(*TD*/*R* + *Tw* × cos*α*/*r*) (9)

where

*r*: radius of the wheel,
*R*: radius of the disk on which the wheel is positioned.

### 3.2. Shape of Magnetic Wheel

**Figure 6:**
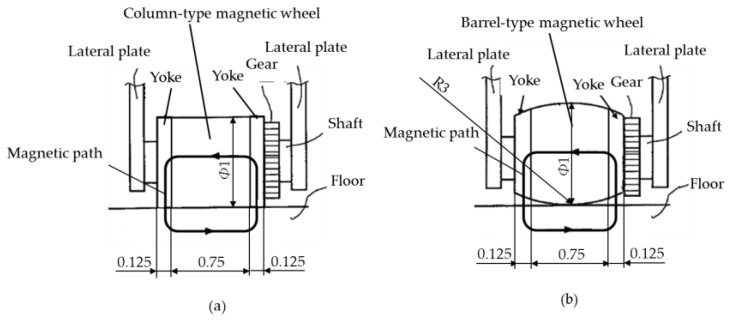
(**a**) Column-type magnetic wheel. The column-type magnetic wheel had a diameter of 1 mm and a length of 0.75 mm, and a disk-type yoke with a width of 0.125 mm was attached to both sides. (**b**) Barrel-type magnetic wheel. The barrel-type magnetic wheel had 1 mm in maximum outer diameter and a length of 0.75 mm, and a disk-type yoke with a width of 0.125 mm was attached to both sides. A radius of 3 mm was attached to the axle direction of the barrel-type magnetic wheel.

Figure 6a,b show two types of magnetic wheels installed in magnetic-wheeled microrobots, column-type and barrel-type, respectively.

In our microrobot prototypes, column-type magnetic wheels of 1 mm in diameter and barrel-type wheels 1 mm in maximum outer diameter were used. A radius of 3 mm was attached to the axle direction of the barrel-type magnetic wheel. SmCo magnet material was adopted, and a disk-type yoke of low carbon steel (SS400) of 0.125 mm width was attached to both sides of the magnet of 0.75 mm in length.

The magnetic wheel used in the slip-evaluation apparatus of Section 3.1. was eight times larger than that of the microrobot. The column-type magnetic wheel was 8 mm in diameter and 8 mm long; a 1mm-wide disk-type yoke was attached to both sides of a 6 mm-long magnet. The barrel-type wheel was 8 times larger as well, and a radius of 24 mm was attached to the axle direction.

**Figure 7:**
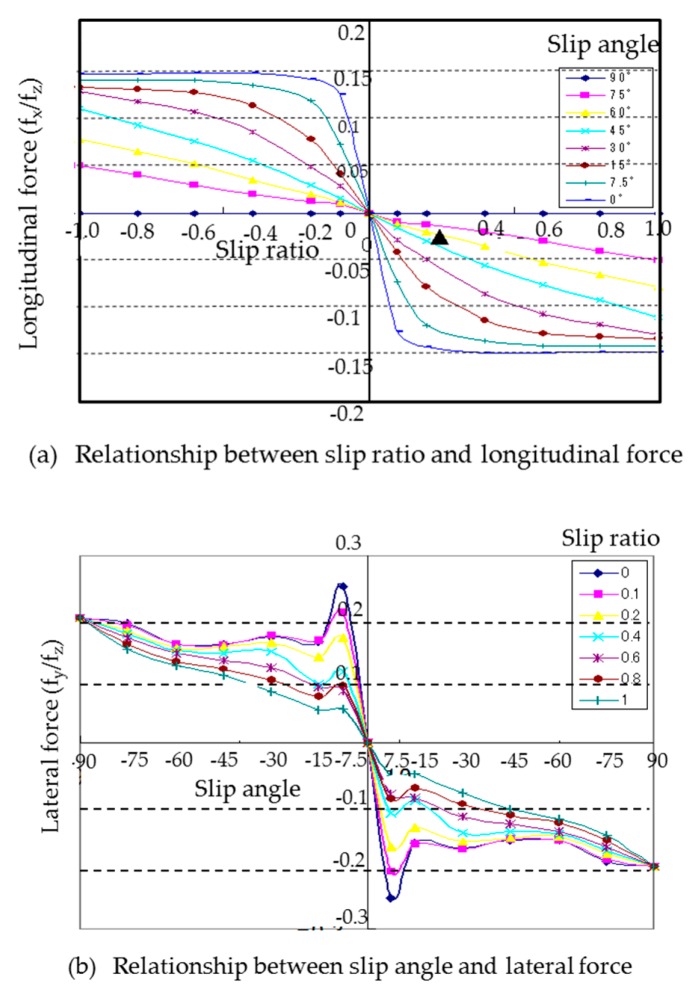
Slip characteristics of column-type magnetic wheel. (**a**) Relationship between slip ratio and longitudinal force where slip angle is changed from 0 degree to 90 degrees. (**b**) Relationship between slip ratio and lateral force where slip ratio is changed from 0 to 1.

Both the column-type and barrel-type magnetic wheels were magnetized in the axial direction with the magnetic path shown in Figure 6 and Figure 7. By magnetizing in the axle direction, even if the magnet wheels rotated, it was possible to rotate efficiently without fluctuating magnetic force.

### 3.3. Measured Slip Characteristics of the Column-Type Magnetic Wheel

An 8 mm-diameter magnetic wheel was positioned such that its axis was parallel to a diameter of a disk of surface roughness 5.5 µm Rmax (slip angle 0 degrees) at 38 mm from the center and the wheel was rotated so that its speed in the rotational direction of the disk was constant. The rotary speed of the disk was such that the slip ratio was zero when the wheel spun at 100 rpm. The frictional forces in the wheel rotatory direction (x direction, longitudinal force) and in the right-angle direction (y direction, lateral force) at the point where the slip ratio was different were calculated with Equation (9); the calculation used the torque-meter measurements for the rotating parts of the wheel and disk by changing the speed of the wheel from 20 rpm to 180 rpm while the disk rotational speed was fixed. Additionally, the slip characteristics of the different slip angles (7.5 degrees, 15 degrees, 30 degrees, 45 degrees, 60 degrees, 75 degrees, 90 degrees) were measured by changing the position of the magnet wheel on the XY stage.

The measured slip characteristics of the column-type magnetic wheel are shown in Figure 7a,b.

The differences in slip characteristics between the tire, which were obtained from Figure 3a,b, and magnetic wheel are as follows:

- In the relationship between the slip ratio and longitudinal force, the maximum longitudinal force, normalized by the vertical force of the magnetic wheel, was approximately 0.15, whereas that of the tire was approximately 0.8. The normalized longitudinal force of the magnetic wheel was flat when the slip ratio was more than 0.4 at a slip angle of zero, whereas that of the tire decreased when the slip ratio was more than 0.2 at a slip angle of zero.
- In the relationship between the slip angle and lateral force, the maximum lateral force, normalized by the vertical force of the magnetic wheel, was approximately 0.25 and decreased to approximately 0.15–0.2 afterwards, but varied with the slip angle, whereas that of the tire was approximately 0.8 and decreased when the slip angle was more than 10 degrees at a slip ratio of zero.

The former result occurred because the magnetic wheel had a rigid surface and a smaller coefficient of friction than the tire. The latter result occurred because when the slip angle became large, the lateral force normalized by the load shifted from a value equivalent to the maximum coefficient of static friction to a value equivalent to a dynamical coefficient; however, line contact between the column-type wheel and disk caused a different speed in the circumferential direction and resulted in unstable movement.

### 3.4. Measured Slip Characteristics of the Barrel-Type Magnetic Wheel

**Figure 8:**
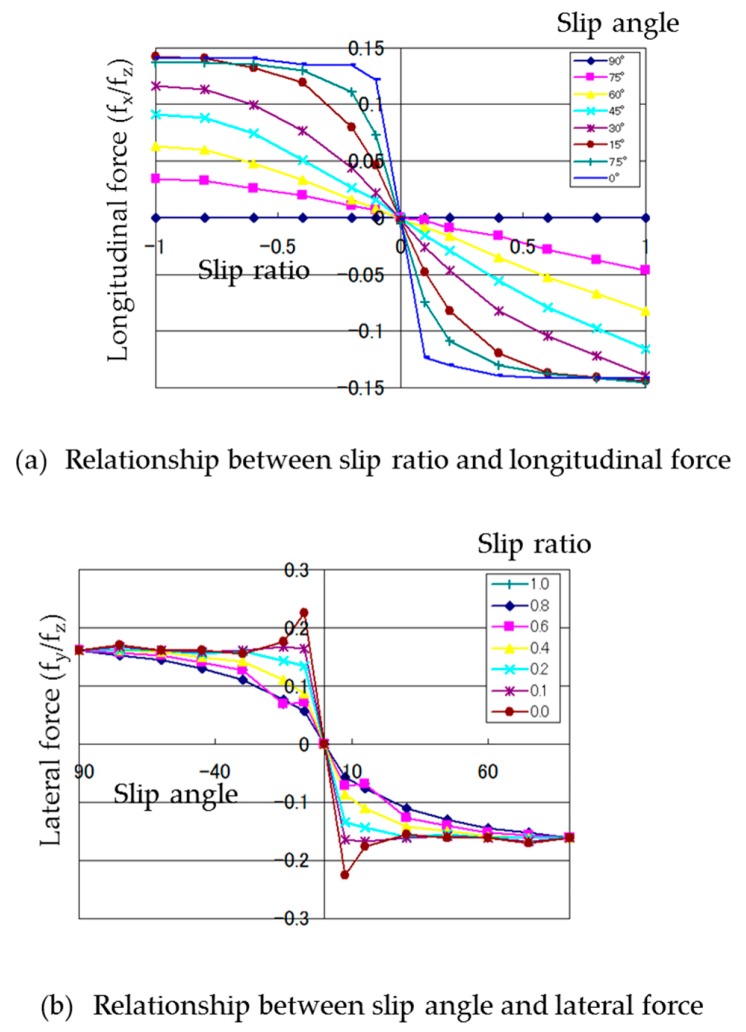
Slip characteristics of barrel-type magnetic wheel. (**a**) Relationship between slip ratio and longitudinal force where slip angle is changed from 0 degree to 90 degrees. (**b**) Relationship between slip ratio and lateral force where slip ratio is changed from 0 to 1.

The slip characteristics of the barrel-type magnet wheel were measured by same method as that described in Section 3.3. The measured slip characteristics of the barrel-type magnetic wheel are shown in Figure 8a,b.

Comparing the slip characteristics of the column-type wheel (Figure 7a,b) to that of the barrel-type (Figure 8a,b), there was not much shape change. However, the convergence level of the barrel-type wheel was slightly smaller than that of the column-type, resulting in slightly easier slip in the relationship between the slip ratio and the longitudinal force (Figure 7a and Figure 8a). As for the relationship between the slip ratio and the lateral force, Figure 7b and Figure 8b show that the peak value and convergence level of the barrel-type wheel were slightly smaller than those of the column-type wheel; the instability improved in the larger part of the slip angle after the peak. This was because the lateral friction force shifted smoothly from static to dynamical friction at the stable contact point between the barrel wheel and the ground. From these results, improvement of driving performance can be expected by using barrel-type magnetic wheels having smooth slip characteristics.

## 4. Simulation Results

Simulations were conducted using the dynamics model in Section 2. Simulation was developed independently using a motion analysis program (SilTools) based on object-oriented language. Features of this simulation included:

- The driving performance of a skid-steered microrobot could be simulated in straight or turning movements.
- Slip and skid occurred in turning because of four fixed-direction wheels. The dynamics model for slips and skids was installed.
- The rotational frequency for a driving wheel could be input instead of torque.
- A driven wheel rotated around its shaft without slipping, following the motion of the main body of the machine. Therefore, a frictional force on the driven wheel in the driving direction did not occur.
- Magnetic attraction could be added in addition to weight as the z-axis force.

### 4.1. Effect of Magnetic Attraction (Distribution of Magnetic Attraction to Driving and Driven Wheels)

**Figure 9:**
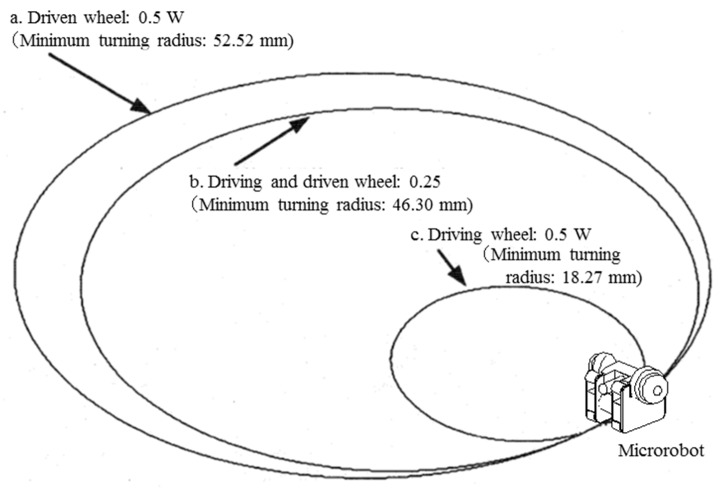
Trajectories (distribution of the magnetic attraction to driving wheels and driven wheels). **a**: Each driven wheel had a magnetic attraction of 0.5 W, and the driving wheels had no magnetic attraction. **b**: Each driven wheel and each driving wheel had a magnetic attraction of 0.25 W. **c**: Each driving wheel had a magnetic attraction of 0.5 W, and the driven wheels had no magnetic attraction.

The magnetic attraction of the driving and driven wheels was simulated when reduction of the gripping force caused by miniaturization was covered by magnetic attraction. Figure 9 shows the trajectories when the distributions of magnetic attraction were changed as follows: the rotations of the right and left driving wheels were set to 100 rpm and 70 rpm, respectively, and the coefficient of maximum static friction was set to 0.3. W is the weight of a single microrobot in the following cases:

a Each driven wheel had a magnetic attraction of 0.5 W, and the driving wheels had no magnetic attraction.
b Each driven wheel and each driving wheel had a magnetic attraction of 0.25 W.
c Each driving wheel had a magnetic attraction of 0.5 W, and the driven wheels had no magnetic attraction.

Figure 9 shows that Case c, in which only the driving wheels were magnetic, had the smallest minimum turning radius of 18.27 mm; Case b, in which all wheels had identical magnetic attraction, had the second-smallest minimum turning radius of 46.3 mm; Case a, in which only the driven wheels were magnetic, had the largest minimum turning radius of 52.52 mm. This result was obtained because the turning characteristics improved with the increase in gripping force between the ground and driving wheels when the driving wheels have magnetic attraction; magnetic attraction of the driven wheels acts as a load and makes it difficult to turn the machine.

### 4.2. Effect of the Magnitude of Magnetic Attraction

**Figure 10:**
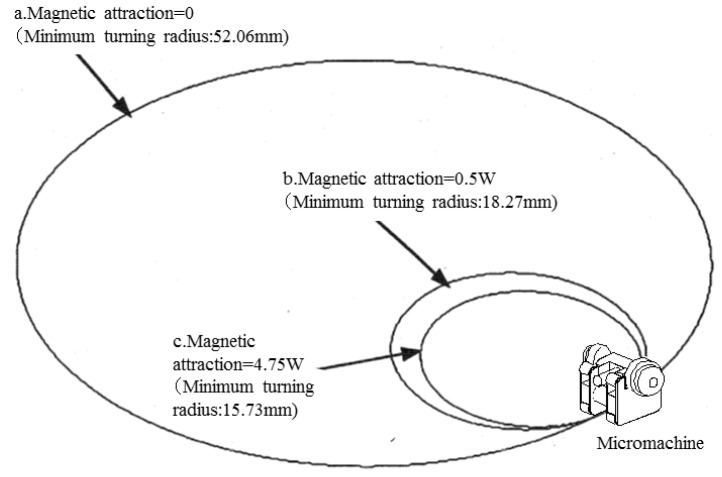
Trajectories (influence of the magnitude of magnetic attraction). **a**: The driving and driven wheels had no magnetic attraction. **b**: Each driving wheel had a magnetic attraction of 0.5 W, and the driven wheels had no magnetic attraction. **c**: Each driving wheel had a magnetic attraction of 4.75 W, and the driven wheels had no magnetic attraction.

Figure 10 shows the trajectories when the magnitude of magnetic attraction was changed as follows: the input rotations of the right and left driving wheels were set to 100 rpm and 70 rpm, respectively, and the coefficient of maximum static friction was set to 0.3, similarly to Section 4.1. W indicates the weight of a single microrobot in the following cases:

a The driving and driven wheels had no magnetic attraction.
b Each driving wheel had a magnetic attraction of 0.5 W, and the driven wheels had no magnetic attraction.
c Each driving wheel had a magnetic attraction of 4.75 W, and the driven wheels had no magnetic attraction.

Figure 10 shows that Case c, in which the driving wheels had a magnetic attraction of 4.75 W, had the smallest minimum turning radius of 15.73 mm; Case b, in which the driving wheels had a magnetic attraction of 0.5 W, had the second-smallest minimum turning radius of 18.27 mm; Case a, in which no wheels had magnetic attraction, had the largest minimum turning radius of 52.06 mm. However, the turning characteristics did not significantly improve compared with the case where the magnetic attraction was l W, even if the total magnetic attraction was as much as 9.5 W.

These results clarified that when the driving performance of a skid-steered microrobot deteriorates because of reduction of the gripping force caused by size reduction, the performance can be improved by adding a magnetic attraction to the driving wheels equivalent to the weight.

### 4.3. Comparison of Slip Characteristics between the Tire and Magnetic Wheel

**Figure 11:**
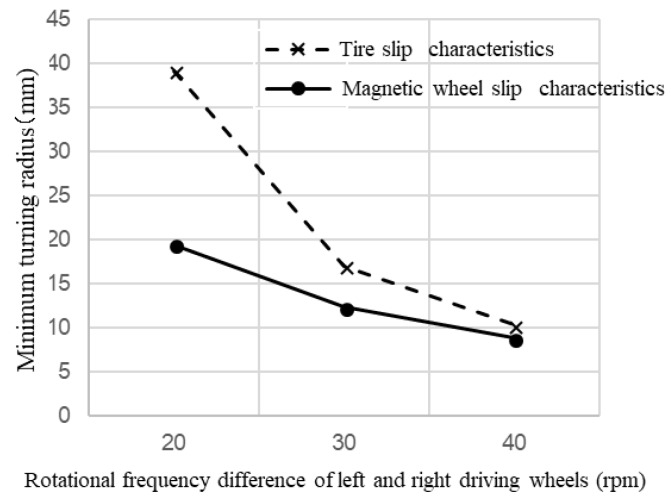
Comparison between tire slip characteristics and magnetic wheel slip characteristics. The dotted line shows the results of tire slip characteristics. The solid line shows the results of magnetic wheel slip characteristics.

The relationship between the minimum turning radius and the difference in rotational frequency of the left and right driving wheels was calculated by simulation. Figure 11 shows the comparison between a tire and magnetic wheel in terms of slip characteristics when the rotational frequency of the left driving wheel was fixed at 100 rpm and the rotation of the right driving wheel was changed between 60, 70, and 80 rpm; the magnetic attractions of the driving and driven wheels were set to 22.05 mN and 2.45 mN. From Figure 11, the magnetic wheel had a smaller minimum turning radius than the tire; this was because of the wheel’s low coefficient of friction. The results indicate that driving performance was improved by more slippery characteristics of the magnetic wheel compared to that of tire.

### 4.4. The Relationship between Microrobot Speed and Rotational Frequency of Driving Wheel

**Figure 12:**
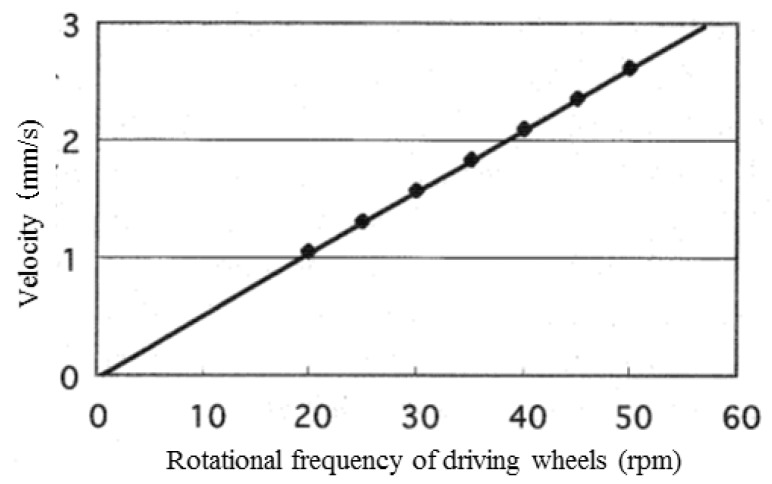
The relationship between microrobot speed and rotational frequency of driving wheel (straight line movement). The points show the calculation results. The solid line shows the regression line derived from these data.

The speed of the microrobot in straight-line movement was calculated by the simulation using magnetic-wheel slip characteristics by making the left and right driving wheel rotational frequencies equal. Magnetic attractions of the driving and driven wheels were set to 22.05 mN and 2.45 mN, respectively. The simulation results in Figure 12 show that microrobot speed was proportional to the driving wheel rotational frequency R (rpm).

The relationship between frequency and speed *V* (mm/sec) is shown in Equation (10) by the regression line derived from these data.

(10) V=0.0524R+0.00007

This proportionality coefficient 0.0524 is equivalent to 1 mm (diameter of wheel) × π/60 (s). In other words, the driving wheels do not slip in straight-line movement.

### 4.5. The Relationship between Minimum Turning Radius and Difference in Rotational Frequency of the Left and Right Driving Wheels

**Figure 13:**
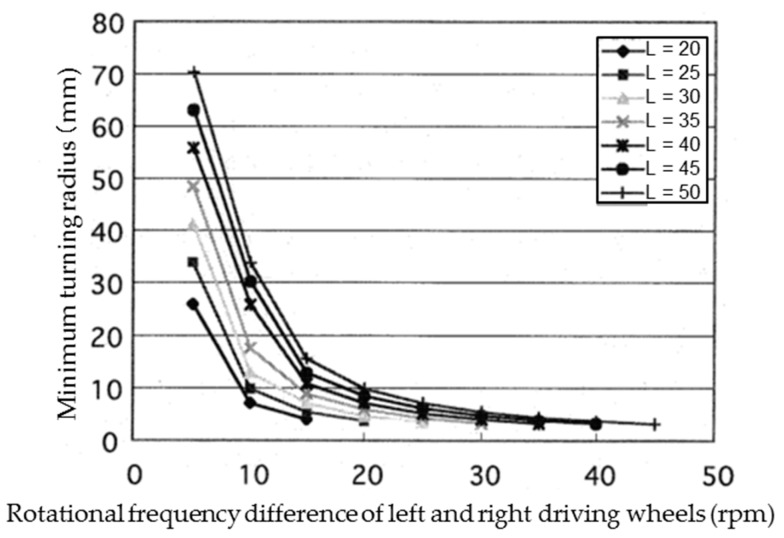
The relationship between the minimum turning radius and the difference in rotational frequency of the left and right driving wheels. The relationship was calculated when the rotational frequency of the left wheel was changed from 20 rpm to 50 rpm in 5-rpm increments.

The microrobot turns with a difference in rotational frequencies of the left and right driving wheels because of the skid-steered method. The relationship between the minimum turning radius and the difference in rotational frequencies of the left and right driving wheels was obtained by the simulation. The relationship was calculated when the rotational frequency of the left wheel was changed from 20 rpm to 50 rpm in 5-rpm increments. Figure 13 shows the simulation results.

**Figure 14:**
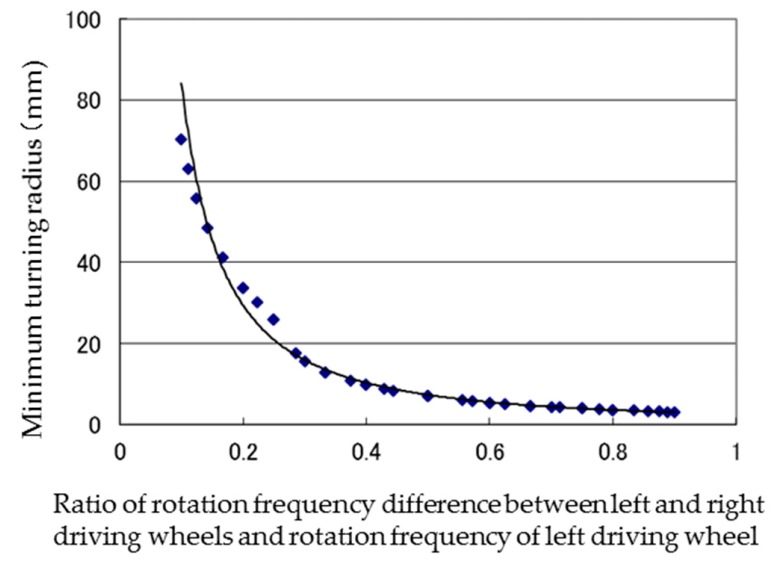
Relationship between ratio of rotation frequency difference between left and right driving wheels and rotation frequency of left driving wheel and minimum turning radius. The points show the calculation results. The solid curve shows the regression curve derived from these data.

Because a similar tendency was observed at all rotational frequencies, the results were replotted (Figure 14) with the horizontal axis set to the ratio of the difference in frequencies to the frequency of the left driving wheel ((frequency of the left driving wheel - frequency of the right driving wheel)/frequency of the left driving wheel).

Figure 14 shows that the minimum turning radius has a one-to-one correspondence to this ratio. The relationship between the minimum turning radius (*Tr*) and the ratio (*r*) is shown in Equation (11) as an approximate equation:

(11) $$T_{r}=2.5735r^{-1.5141}.$$

This shows that a stable turning movement can be realized by the skid-steered method by using magnetic wheels.

## 5. Prototype and Evaluation of a Magnetic-Wheeled Microrobot

### 5.1. Outline of the Prototype

**Figure 15:**
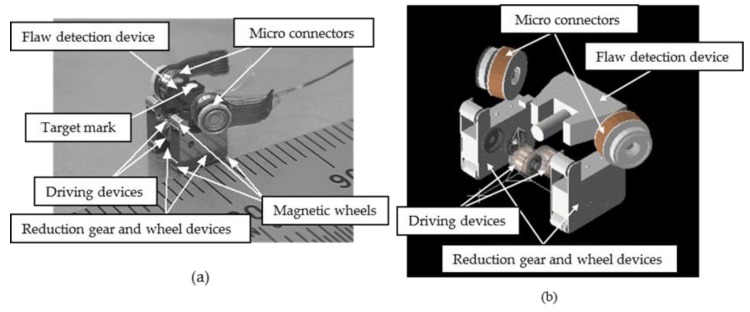
(**a**) Photograph of the microrobot prototype, (**b**) exploded view of the microrobot prototype. Configuration of a prototype of magnetic wheeled microrobot. It consisted of four functional devices: driving devices, reduction gear and wheel devices, micro-connectors, and a flaw-detection device.

Figure 15 shows the configuration of a prototype of a magnetic-wheeled microrobot.

It consisted of four functional devices: driving devices, reduction gear and wheel devices, micro-connectors, and a flaw-detection device. As shown in Figure 15, the two reduction-gear and wheel devices were positioned on both sides of the two driving devices. The flaw detection device was positioned on top of the reduction gear and wheel devices, and two micro-connectors were placed on both sides of the flaw detection device. This simple structure facilitated the assembly process. Unlike a conventional machine in which parts are added to a frame, each functional device functioned as its own frame. The combination of several functions in one functional device enables the miniaturization of the machine. The wheeled method was used for driving because efficient high-speed movement could be realized. Previous studies have concluded that the wheeled method is not suitable for a microrobot because the gripping force between the wheel and ground is low due to its light weight. However, we solved this problem using magnetic wheels. The developed prototype had six magnetic wheels to climb up a vertical wall, as shown in Figure 15. The prototype’s size was 9 mm in width, 5 mm in length, 6.5 mm in height, and its weight was 0.5 g. The microrobot could be automatically connected and disconnected to adjacent microrobots using micro-connectors with electromagnets.

### 5.2. Horizontal Driving Performance Evaluation System

**Figure 16:**
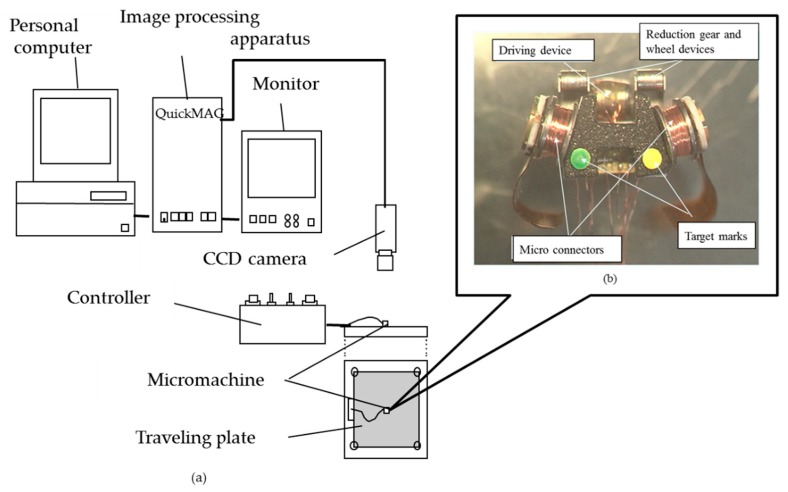
(**a**) Configuration of turning characteristics evaluation system. The system consisted of a traveling plate, a controller, a CCD camera, an image-processing apparatus, a monitor, and a personal computer. (**b**) Top view of microrobot. The green and yellow marks were target marks for position detection. The marks were 1 mm in diameter, 3.66 mm apart and were located 1.55 mm behind the microrobot center.

The configuration of the system for evaluating horizontal driving performance is shown in Figure 16a. Fluorescent green and yellow marks were attached to the top black surface of the prototype for easy extraction, as shown in Figure 16b.

The system consisted of a traveling plate made of magnetic SUS 430 (surface roughness: 1µm Rmax), a controller (Original made, Tokyo, Japan), a CCD camera (TN411: made in ELMO, ELMO, Nagoya, Japan), an image-processing apparatus with a monitor (QuickMAG: made in OKK, Itami, Japan), and a personal computer(Endeavor Pro400: made in EPSON DIRECT, Japan).

The marks were 1 mm in diameter, 3.66 mm apart and were located 1.55 mm behind the microrobot center. They were used to measure the machine position and driving direction. The movement of the mark could be detected with the video rate of 60 frames/s. Before measuring, calibration was done using a calibration gauge with a precisely arranged array of color marks, and the measurement range was determined. Then, initial settings such as color extraction of the marks on the microrobot and measurement time were recorded, and measurements were performed on the moving microrobot. The turning characteristics of the microrobot were evaluated by analyzing the traveling trace and speed of the microrobot during turning using the turning-characteristics evaluation system.

### 5.3. Performance-Measurement Results for Straight-Line Movement

**Figure 17:**
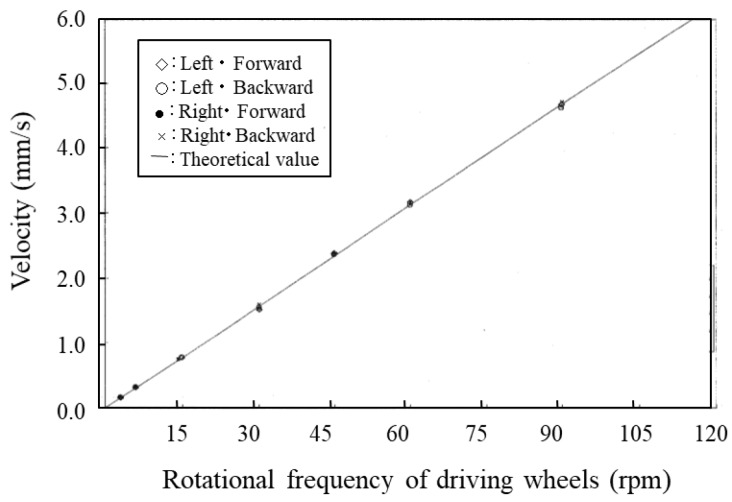
Relationship between the speed of left and right target marks and rotational frequency of driving wheel in forward and backward movement. The marks show the measurement results and the solid line shows the theoretical line.

Straight-line movement of the microrobot prototype on the traveling plate was measured by the horizontal driving performance-evaluation system described in Section 5.2. while the left and right driving devices had the same rotational frequency. The trajectories of the microrobot prototype in both forward and backward movement were measured at various rotational frequencies of driving wheels. Forward and backward movements were measured once in each case with seven driving-wheel rotational frequencies: 3 rpm, 6 rpm, 15 rpm, 30 rpm, 45 rpm, 60 rpm, and 90 rpm. The measurements were performed at the same position on the traveling plate because of elimination of the influence of the wiring. The microrobot was moved forward for approximately 10 seconds and was moved backward from a stopped position, again for approximately 10 seconds. Figure 17 shows the resulting relationship between the speeds of the left and right marks and the driving-wheel rotational frequency in forward and backward movement. In addition, a straight line in Figure 17 shows the theoretical speed. Figure 17 shows that there are few differences between the theoretical and actual speeds of the left and right marks as well as forward and backward movement. This result is consistent with the simulation results in Section 4.4.

### 5.4. Comparison of Turning Performance between Experiment and Simulation

**Figure 18:**
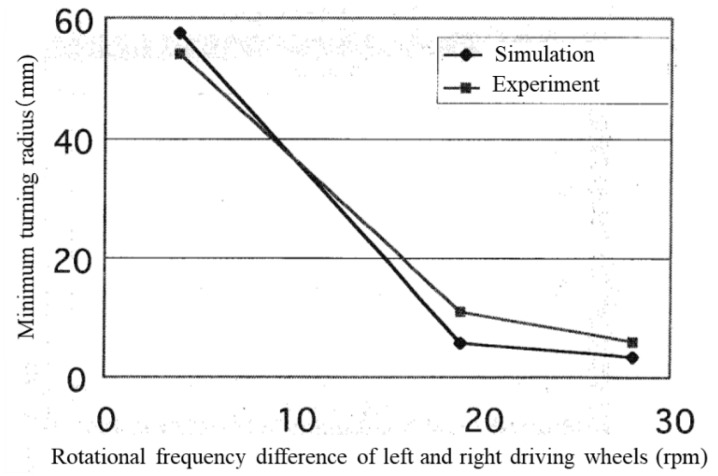
Relationship between the minimum turning radius and the difference of the rotational frequencies of left and right driving wheels of experiment and simulation. The circle marks show the simulation results and square marks show the experimental results.

Figure 18 shows the relationship between the minimum turning radius and the difference in rotational frequency between the left and right driving wheels in the experiment and simulation when the rotation of the right driving wheel was 29, 14, and 5 rpm while the left driving wheel was 33 rpm. The experimental and simulation results of turning performance were consistent according to the Figure 18.

## 6. Discussion

### 6.1. Measurement Results of Forward and Backward Turning Movement

**Figure 19:**
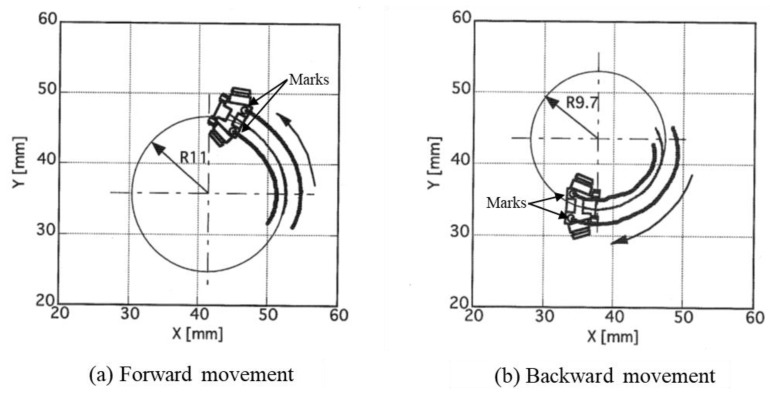
Turning trajectories. (**a**) The trajectories of forward movement. (**b**) The trajectories of backward movement. The three trajectories are those of the two target marks and the center of the microrobot. The circle drawn by a thin line is a circle of the minimum turning radius obtained from the trajectory.

Figure 19 shows the trajectories and minimum turning radii of the microrobot when the input rotational frequencies of the left and right driving wheels were set to 14.4 and 33.3 rpm.

Figure 19a,b show the trajectories of forward and backward movements, respectively. The three trajectories are those of the two target marks and the center of the microrobot. Comparison of the figures shows that forward movement was smoother than backward movement and that the minimum turning radius of backward movement was slightly smaller than that of forward movement. We discuss the reasons below.

**Figure 20:**
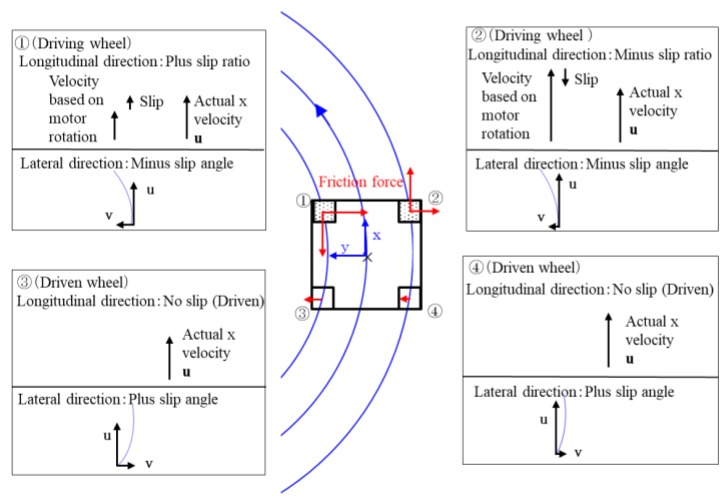
State of each wheel and friction force in forward movement. The red lines show the frictional forces acting on each wheel.

Figure 20 shows the state of, and longitudinal and lateral frictional forces on, each wheel in the case of forward turning to the left.

In forward movement, the front wheels were driving wheels and the rear wheels were driven wheels; therefore, the microrobot had front-wheel drive. The actual speed of driving wheel ① became faster than the input rotational speed because of slip. In contrast, the actual speed of driving wheel ② became slower than the input rotational speed because of slip. The driven wheels had no longitudinal slip because of the assumption of the model. Each wheel had a different radius from the instantaneous center of rotation. As for the lateral direction, the front wheels had a negative skid and the rear wheels had a positive skid. Friction acts in the opposite direction of slip by the definition of slip and friction force. Although the left-front wheel (①) rotated in the direction of travel, it worked as a net braking force because of slip. The front wheels skidded to the left of the line during turning, and reverse forces acted on the wheels from the ground as a reaction. Similarly, the rear wheels skidded to the right of the line during turning, and reverse forces acted on the wheels from the ground as a reaction. The moment acting on a microrobot was determined from these frictional forces and their distance from its center of gravity. The longitudinal and lateral forces corresponding to the slip ratio and slip angle respectively, acted on each wheel according to the slip characteristics shown in Figure 8a,b for the barrel-type magnetic wheel. In the case of forward movement, a rear wheel was easy to skid because the magnetic attraction of a driven wheel was approximately 1/10 of the attraction of the driving wheel, and only the small frictional force acted. However, the front wheel pulled a rear wheel, and achieved a stable pivot-like movement because it had a large magnetic attraction and sufficient gripping force.

**Figure 21:**
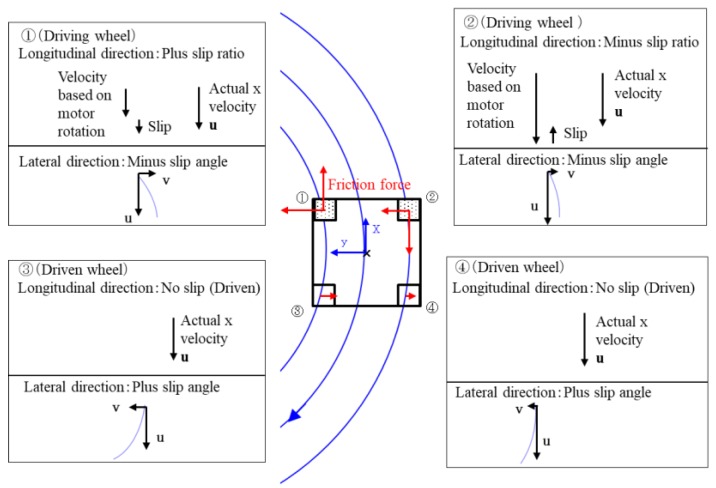
State of each wheel and friction force in backward movement. The red lines show the frictional forces acting on each wheel.

Figure 21 shows the state of, and longitudinal and lateral frictional forces on, each wheel in the case of backward turning to the right.

When moving backward, the driving wheels were in the rear and the front wheels were driven wheels; therefore, the microrobot has rear-wheel-drive. Similar to the forward movement, the actual speed of driving wheel ① became faster than the input rotational speed because of slip. The actual speed of driving wheel ② became slower than the input rotational speed because of slip. The driven wheels had no slip in the longitudinal direction because of the assumption of the model. Each wheel had a different radius from the instantaneous center of rotation. In the lateral direction, the front wheels, located in the rear in this traveling direction, had negative skid, and the rear wheels, located in front for this traveling direction, had positive skid. Friction acts in the direction opposite to slip from the definitions of slip and friction force. Although the left-front wheel (①) rotated in the traveling direction, it worked as a breaking force because of slip. The front wheels (① and ②) skidded to the left of the line during turning, and reverse forces acted on the wheels from the ground as reactions. Similarly, the rear wheels (③ and ④) skidded to the right of the line during turning, and reverse forces acted on the wheels from the ground as reactions. The moment acting on the microrobot was determined from these frictional forces and their distances from the center of gravity of the microrobot. The longitudinal and lateral forces corresponding to the slip ratio and angle, respectively, acted on each wheel following the slip characteristics in Figure 8a,b for the barrel-type magnetic wheel. In addition, in backward movement, rear wheels (③ and ④), located forward for this direction of travel, skidded easily when pushed by a driving wheel (① and ②), located in the back for this direction of travel, because the magnetic attraction of a driven wheel was only approximately 1/10 of that of a driving wheel, and only a small frictional force acted. Backward movement was slightly unstable compared to forward movement because the direction of travel of the driven wheels, located in front of the line, did not necessarily coincide with that of the driving wheels. Moreover, the minimum turning radius of the backward movement was smaller than that of forward movement because the rear wheels (③ and ④), located in front of the line, skidded, largely by being pushed by the front wheels (① and ②), with their strong magnetic attraction and sufficient gripping force. These were the same relationships as those between front-wheel and rear-wheel drives of a car.

### 6.2. Comparison of Turning Performance between Column-Type and Barrel-Type Magnetic Wheels

**Figure 22:**
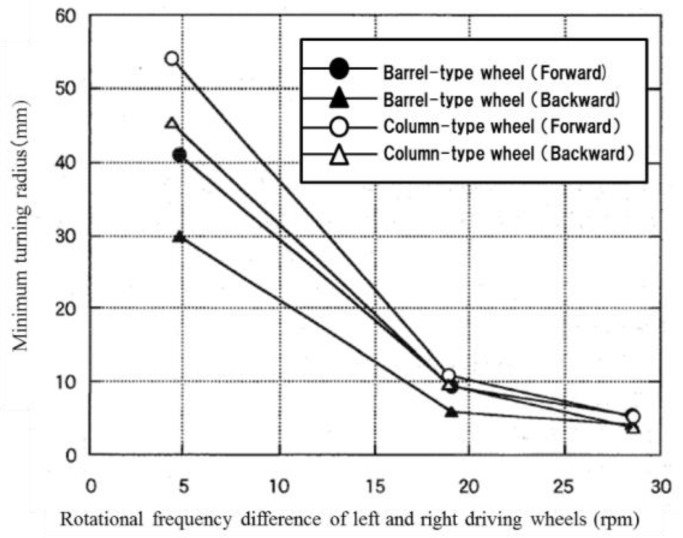
Relationship between the minimum turning radius and the difference of the rotational frequencies of left and right driving wheels (comparison of barrel-type wheel and column-type wheel). The circle marks show the results of forward movement and triangle marks show the results of backward movement. The black marks show the results of barrel-type wheel and white marks show the results of column-type wheel.

The turning performance of the microrobot with barrel-type magnetic wheels was compared to that of one with column-type magnetic wheels. The minimum turning radii were measured when the difference in rotational frequencies of the left and right driving wheels was 4.8 rpm, 19.4 rpm and 28.5 rpm in forward and backward movement. Figure 22 shows the relationship between the minimum turning radius and the difference in rotational frequencies of the left and right driving wheels for a microrobot with barrel-type magnetic wheels compared with one with column-type magnetic wheels.

First, the turning radius of backward movement was smaller than that of forward movement for both the barrel-type and column-type magnetic wheels. This result was a result of the performance difference between front-wheel and rear-wheel drives as described in Section 6.1. In addition, the minimum turning radius of the barrel-type wheel was smaller than that of the column-type wheel for both forward and backward movements when the differences in rotational frequencies were small; there was not much difference between the wheel types when the difference of rotational frequency was large. In other words, the effect of a barrel-type magnetic wheel was apparent when the turning radius was large; however, the effect disappeared when the turning radius was small because smooth turning could not be achieved due to the unstable slip characteristics between the column-type wheel and the ground. The influence of unstable slip characteristics between the column-type wheel and the ground decreased with a large friction force when the difference of rotational frequency was large.

## 7. Conclusions

The dynamics of a skid-steered wheeled microrobot with magnetic attraction and slip was derived to develop a wheeled microrobot. The slip characteristics of the magnetic wheel were measured, and the turning characteristics of a microrobot using this wheel were analyzed. In addition, a skid-steered magnetic-wheeled microrobot of 9 mm in width, 5 mm in length, and 6.5 mm in height was developed, and its turning characteristics were measured. The following conclusions can be drawn:

- We clarified that driving performance can be improved by using magnetic wheels and giving magnetic attraction equivalent to self-weight to the driving wheels.
- The slip characteristics of column-type and barrel-type magnetic wheels were measured; the results revealed that magnetic wheels do not have the nonlinearity of rubber (tires) because of their hard surfaces.
- A simulator was developed for the slip characteristics of a magnetic wheel and magnetic attraction. The simulation results were consistent with the experimental results.
- Driving performance was different between forward movement and backward movement when turning. It was clarified that the reason was the difference between front-wheel drive and rear-wheel drive.
- The barrel-type wheel had better drive performance than the column-type wheel because of smoother slip characteristics. This tendency was large with a small difference of rotational frequencies of the left and right wheels and decreased with a large difference.
